# What potential has tobacco control for reducing health inequalities? The New Zealand situation

**DOI:** 10.1186/1475-9276-5-14

**Published:** 2006-11-02

**Authors:** Nick Wilson, Tony Blakely, Martin Tobias

**Affiliations:** 1 Department of Public Health, Wellington School of Medicine and Health Sciences, Otago University, PO Box 7343 Wellington South, New Zealand; 2 Ministry of Health, PO Box 5013, Wellington, New Zealand

## Abstract

In this *Commentary*, we aim to synthesize recent epidemiological data on tobacco and health inequalities for New Zealand and present it in new ways. We also aim to describe both existing and potential tobacco control responses for addressing these inequalities.

In New Zealand smoking prevalence is higher amongst Māori and Pacific peoples (compared to those of "New Zealand European" ethnicity) and amongst those with low socioeconomic position (SEP). Consequently the smoking-related mortality burden is higher among these populations. Regarding the gap in mortality between low and high socioeconomic groups, 21% and 11% of this gap for men and women was estimated to be due to smoking in 1996–99. Regarding the gap in mortality between Māori and non-Māori/non-Pacific, 5% and 8% of this gap for men and women was estimated to be due to smoking. The estimates from both these studies are probably moderate underestimates due to misclassification bias of smoking status. Despite the modest relative contribution of smoking to these gaps, the absolute number of smoking-attributable deaths is sizable and amenable to policy and health sector responses.

There is some evidence, from New Zealand and elsewhere, for interventions that reduce smoking by low-income populations and indigenous peoples. These include tobacco taxation, thematically appropriate mass media campaigns, and appropriate smoking cessation support services. But there are as yet untried interventions with major potential. A key one is for a tighter regulatory framework that could rapidly shift the nicotine market towards pharmaceutical-grade nicotine (or smokeless tobacco products) and away from smoked tobacco.

## Background

As for other countries, the distribution of disease burden in New Zealand is far from equal [1-4]. In particular, there are much higher rates of premature death and of serious chronic diseases for the poorest New Zealanders, for Māori (the indigenous people of New Zealand), and for Pacific peoples living in this country. Māori adult mortality rates are at least twice those of non-Māori in New Zealand. Such inequitable patterns are a concern for the government and the health sector for the ethical reason of ensuring justice but also because the New Zealand Government is committed to improving Māori health under the obligations of the Treaty of Waitangi (signed in 1840 between the British Crown and Māori chiefs). In particular, Article Three of this Treaty translates into an obligation for Crown agencies to work to ensure that Māori citizens enjoy the same rights as others, including the right to good health. Section 8 of the New Zealand Public Health and Disability Act (2000), specifically requires health services to recognize the principles of the Treaty of Waitangi [5].

Other arguments for reducing health inequalities are less prominent in the New Zealand discourse, but include the benefits of enhancing overall public health and social cohesion and the resultant economic benefits. The latter may arise from preventing premature deaths among workers and reducing productivity losses associated with worker illness.

Given these issues, we aimed to synthesize recent epidemiological data on tobacco and health inequalities for New Zealand, and to present it in new ways. We also aimed to describe existing and potential tobacco control responses for addressing these inequalities. Our focus is on socioeconomic and ethnic health inequalities, and we leave other inequalities (eg, gender, regional) to other forums.

## Social and ethnic patterning of tobacco use in New Zealand

Many international studies provide strong evidence that smoking prevalence is patterned by socioeconomic position (SEP) [6-10], and the same is true in New Zealand [1,11,12]. There is also evidence that smoking prevalence in this country has become more strongly patterned by SEP over time [11,12]. One reason for this is that the uptake of smoking by young people has declined more steeply amongst those in the highest income level over recent decades [13]. Māori and Pacific peoples have a higher smoking prevalence than non-Māori/non-Pacific, partly reflecting relative socioeconomic disadvantage.

Another reason for the increase in the SEP patterning of smoking over time is probably because the quit rates among higher-SEP New Zealanders have increased more than for other groups [13]. The difference in quit rates by SEP may be due to such factors as: (i) the impact of educational level on knowledge of tobacco risks and motivation and knowledge of how to quit; (ii) economic barriers to quitting technologies (eg, the price of nicotine replacement therapy was fairly high until recently and there are still cost barriers for some pharmaceutical aids such as bupropion); and (iii) differential levels of social and other support for quitting. With the latter for example, second-hand smoke exposure is higher in low-income groups [14] and for Māori [14,15]. Also, the first major smokefree law (in 1990) benefited office workers more than factory workers in terms of reducing exposure to second-hand smoke [16].

## Studies on tobacco and health inequalities in New Zealand

### Lung cancer as a marker of historic tobacco exposure

**Figure 1:**
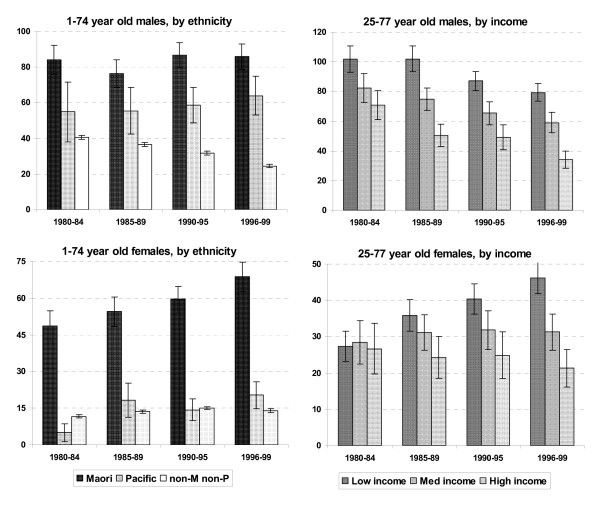
Age standardised lung cancer mortality rates in New Zealand by ethnicity and household income, males and females (per 100,000 population). **Source: **Data derived from: [17]. The bars indicate 95% confidence intervals. Note the different age range for ethnicity and household income. The ethnic mortality rates were calculated using adjustment factors (from the NZCMS) for historic undercounting of Māori and Pacific deaths [2, 3], and the income mortality rates were calculated directly from linked census-mortality data. Rates by household income are standardised or both age and ethnicity. *Ethnicity definitions*: The definition of ethnicity progressively changed from fractionated ethnic origin in the 1981 census (eg, 7/8 European, 1/8 Māori), to multiple self-identified ethnicity in 1996 elicited by the question: "Tick as many circles as you need to show which ethnic group(s) you belong to". This change in the question and secular trends in how people viewed their own ethnicity led to a disproportionate increase in the Māori population (than expected on the basis of demographic projections alone). However, trends in mortality rates shown above are largely unaffected, as the numerators have been adjusted to be consistent with the denominators.

Lung cancer is the cause of death that most directly reflects the (historic) burden of smoking. Figure 1 shows lung cancer mortality rates by ethnicity and household income, for the 1980s and 1990s, as calculated from the New Zealand Census-Mortality Study (NZCMS) that uses linked census and mortality datasets covering millions of person-years of observation [17]. Lung cancer mortality rates among Māori were over four times the non-Māori/non-Pacific rate for women and over three times for men (for 1996–1999). The rates for Pacific people were also relatively high (at over 2 times for men and 1.4 times for women, compared to non-Māori/non-Pacific). Over the same 1981–1999 time period, the inequality in male lung cancer mortality rates by household income persisted despite a decline in deaths in all income groups. However, in women there was a large increase among the low-income group compared to a decrease among the high-income group. Over this time period there was also an overall *increase *in ethnic inequalities in mortality rates from lung cancer (in both men and women). The authors of this study concluded that these inequalities will probably widen in future decades – unless there is concerted public health action. All these patterns are consistent with differently phased tobacco epidemics [18] by ethnicity and SEP, resulting in changing inequalities in lung cancer over time.

The very large inequalities in lung cancer mortality by ethnicity are probably greater than would result alone from historically (still large) differences in smoking prevalence, pointing to other independent, and likely interacting, risk factors. These may include such factors as: varying passive smoking exposure [14,15], environmental pollution exposure [19] and hazardous occupational exposure such as from asbestos [20]. Diet may also be relevant to this differential (eg, given evidence around fruit intake lowering lung cancer risk [21]) and so might genetics given some New Zealand evidence for variability in nicotine metabolism by ethnicity [22].

Differential survival, due to differential access to care and more advanced stage at presentation will also contribute to ethnic inequalities in lung cancer mortality. Māori are more likely than non-Māori to have lung cancer identified at a later stage and have a lower survival rate after diagnosis [23]. Possible factors involved include access to specialised cancer services and the quality of care received [24].

### The contribution of active tobacco smoking to mortality burden within ethnic and socioeconomic groups, and the mortality gap between these groups

The NZCMS includes active smoking data for the 1981–84 and 1996–99 cohorts, allowing direct estimations of the active smoking-related burden within and between social groups. The measure of smoking is simply "never", "ex-" and "current" smokers, meaning there will be inevitable misclassification biases of smoking that probably lead to modest underestimates of the contribution of active smoking.

Table 1 shows population-attributable risk percents (PAR%) for 45–74 year olds in 1996–99 from NZCMS output. They are the percentage reduction in all-cause mortality that might be expected if, in a counterfactual world, all people who were either current or ex-smokers had actually been "never" smokers. Because of slightly (and necessarily) different methods between the ethnic and educational group analyses (see footnotes to Table 1), they are not fully comparable. Nevertheless, they do robustly point to the following conclusions:

**Table 1:** The estimated percentage decrease (population-attributable risk percent (PAR%)) in 45–74 year old mortality rates during 1996–99 had all current and ex-smokers actually been never smokers

	**Men 1996–99**				**Women 1996–99**			
	
***Within educational group †***	**PAR% in total population**	**PAR% within educational group**			**PAR% in total population**	**PAR% within educational group**		
				
		**Nil**	**School**	**Post-school**		**Nil**	**School**	**Post-school**
(ii) All current and ex-smokers become never smokers in each educational group (ie, historically smokefree).	26%	29%	26%	23%	25%	27%	24%	23%
	
***Within ethnic group ‡***	**PAR% in total population**	**PAR% within ethnic group**			**PAR% in total population**	**PAR% within ethnic group**		
				
		**Māori**	**nMnP**			**Māori**	**nMnP**	

(ii) All current and ex-smokers become never smokers in each ethnic group (ie, historically smokefree).	33%	21%	36%		28%	25%	28%	

† Source: Table 4 from [75].

‡ Source: PAR% calculated from data in: [27].

NB: The educational PAR% estimates are calculated using Poisson rate ratios adjusted for age and ethnicity, whereas the ethnic PAR% estimates are based on age-standardised mortality rates.

nMnP – non-Māori non-Pacific (ie, mainly "New Zealand European" ethnicity).

See the footnotes to Figure 1 for ethnicity definitions.

• active smoking is a major contributor to all-cause mortality in all educational and ethnic groups,

• about a quarter of 45–74 year old all-cause mortality in each educational group is due to active smoking. This figure is slightly higher in lower educational groups, and slightly less in higher educational groups,

• about a third of 45–74 year old all-cause mortality among non-Māori/non-Pacific is due to smoking. This figure is slightly higher among males, and slightly less among females,

• a fifth to a quarter of 45–74 year old all-cause mortality among Māori is due to smoking.

These above estimates for Māori are less than expected based on previous Ministry of Health estimates that a third of *all *Māori deaths (not just 45–74 year olds where a greater proportion of deaths will be due to smoking than at other ages) are due to tobacco [25]. There are two key reasons why the more recent Ministry of Health estimates for Māori are likely overestimates. First, other recent work from the NZCMS finds that the relative risk of death associated with tobacco use varies by ethnic group and over time [26]. All-cause rate ratios (RRs) for mortality associated with smoking were significantly greater within non-Māori/non-Pacific than within Māori: 2.22 compared to 1.51 respectively for men, and 2.20 compared to 1.45 respectively for women (for 1996–99). One of the likely reasons for this rate ratio heterogeneity is the greater role of competing non-tobacco causes of mortality among Māori and Pacific peoples. But other factors may also be relevant eg, different patterns of what cigarettes are used and how they are smoked. Second, the Ministry of Health estimates have used the standard WHO/Peto methodology whereby lung cancer mortality rates are used to estimate the total mortality impact of smoking. However, as mentioned above, Māori lung cancer mortality rates are higher than would be expected on the basis of tobacco smoking alone, which would lead the WHO/Peto method to overestimate the total tobacco-related mortality burden among Māori.

What of the contribution of smoking to **gaps **in mortality between ethnic and socioeconomic groups? Poisson regression analyses adjusting for smoking reduced the all-cause mortality RRs for men with nil educational qualifications compared with men with post-school qualifications from 1.34 to 1.29 in 1981–84 and from 1.31 to 1.25 in 1996–99. This equated to 16% and 21% reductions in relative inequalities respectively. The equivalent results for women were 3% and 11% reductions in relative inequalities for these time periods. Such higher mortality rates for men and women with poorer education were due to the impact of smoking on cardiovascular, cancer and respiratory deaths. The patterns identified in this study were considered to reflect the historically differential phasing of the tobacco epidemic by sex and SEP.

The most recent NZCMS study on smoking examined its contribution to ethnic inequalities in mortality [27]. It found that the apparent contribution of smoking to mortality differences between Māori and non-Māori/non-Pacific was greatest for women in 1996–99 (8% reduction in standardised rate difference), and had increased from 1981–84 to 1996–99 for both men (from -1% to 5%) and women (from 3% to 8%). That is, the contribution of smoking to ethnic gaps (in percentage terms) is notably less than for socioeconomic gaps. But a fuller understanding of this requires also considering the actual underlying mortality rates.

**Figure 2:**
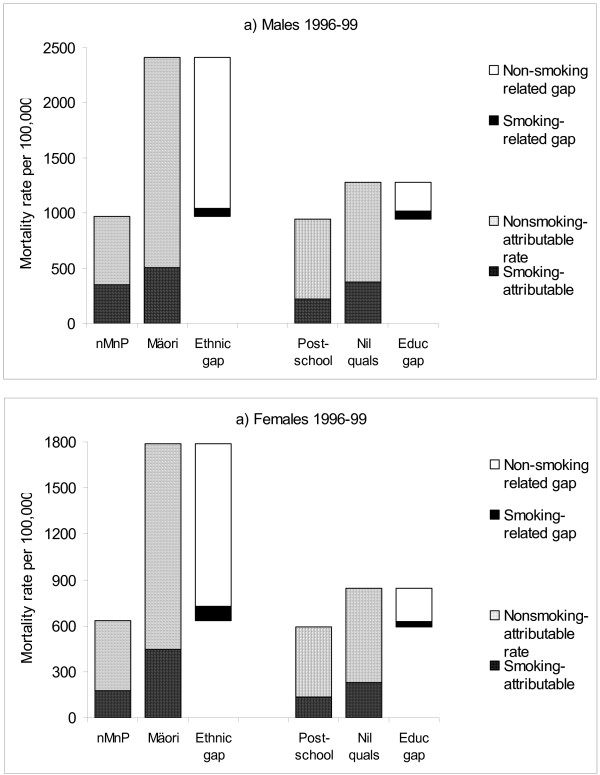
The contribution of active tobacco smoking to 45–74 year old age-standardised mortality rates, and gaps in mortality rates, in 1996–99, by ethnicity and education (with the latter as a marker for SEP). **Sources: **Data derived from: [75] and [27]. nMnP – non-Māori non-Pacific (ie, mainly "New Zealand European" ethnicity). See the footnotes to Table 1 for ethnicity definitions.

Figure 2 attempts to pull together the above findings for 1996–99, and addresses the need to consider absolute mortality rates and absolute differences in mortality rates (as well as relative risks and percentage contributions). It shows actual mortality rates by ethnicity and education (partitioned by the proportions estimated to be smoking- and non-smoking related). A floating column representing the gap in mortality rates is included (again partitioned into smoking and non-smoking-related components). The figure should be considered indicative only. There are unavoidable differences in methodology between: the ethnic versus educational analyses as stated above; the determination of PAR% within ethnic and socioeconomic group versus the percentage contributions to gaps between ethnic and socioeconomic groups; and standardisation versus regression methodologies for different components of analysis behind the figure. Nevertheless, there are a number of robust findings:

• mortality rates for Māori are 2–3 times greater than non-Māori/non-Pacific, compared to an approximately 40% higher mortality for people with no qualifications compared to post-school qualifications;

• in *absolute *terms, the mortality rate attributable to smoking among both Māori and less educated groups is considerably greater than among non-Māori/non-Pacific and post-school educated people, respectively – a different perspective from considering the PAR% estimates in isolation;

• in *absolute *terms, the gap in mortality rates between ethnic groups attributable to smoking is as great or greater than between educational groups – a different perspective from considering just the *percentage *contribution to gaps.

As mentioned already, the estimates above are likely to be modest underestimates due to likely non-differential misclassification bias of smoking status. The analyses did not include the impact of exposure to second-hand smoke, which is more common among Māori and lower socioeconomic groups [14,15]. This would mean that percentage contributions of active and passive smoking combined to mortality are probably greater than given above. Figure 2 also clearly demonstrates that ethnic gaps in mortality not explained by smoking are much greater than socioeconomic gaps in mortality not explained by smoking. This points to other determinants of health (eg, differential access to health services, racism) that must be more important for ethnic inequalities than socioeconomic inequalities in health.

## What can be done to reduce health inequalities from tobacco in New Zealand?

**Figure 3:**
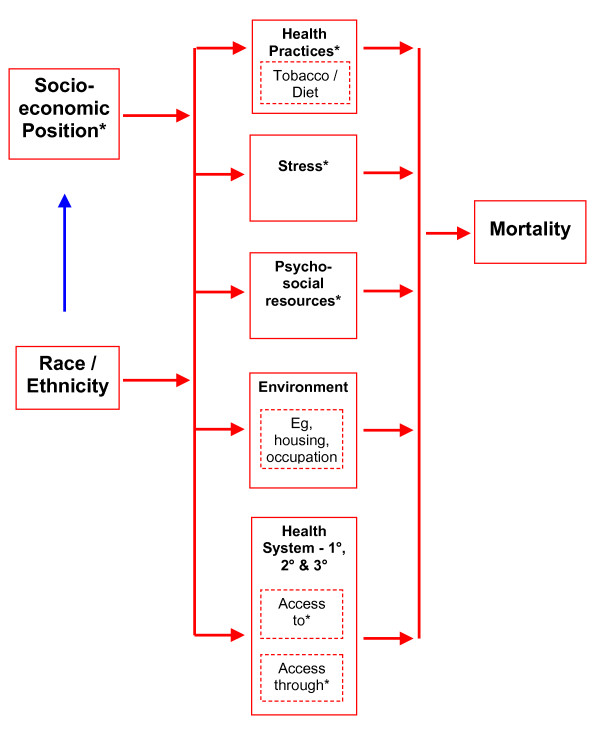
Simplified causal/intervention model for pathways between ethnicity and socioeconomic position to mortality. * Direct interpersonal racism and institutional racism probably has a diffuse impact on many causal processes represented by this diagram, including the unequal distribution of socioeconomic resources, the quantity and quality of "stress" and "psychosocial resources", "access to/access through the health system", and patterns of drug use – including smoking. There are New Zealand specific data on racism and health and racism and smoking [33, 34].

Despite the apparently modest relative size of these tobacco-related gaps, their absolute magnitude means that eliminating them would still be very worthwhile. Reducing these tobacco-related gaps may also be achievable given the strong evidence base for traditional tobacco control interventions and the evidence supporting their cost-effectiveness [28,29]. Nevertheless, many other options for reducing health inequalities could still be progressed at the same time, including: more equitable income redistribution in New Zealand [30], improvements in educational levels, housing policies, policies to reduce unemployment and improving access to and through health services for low-income New Zealanders (eg, see Figure 3). Community-level interventions to enhance trust and promote safe environments have also been suggested for reducing inequalities and lowering smoking – given evidence that low social capital may be independently associated with higher smoking prevalence [31]. Improving work conditions may also be relevant to reducing tobacco use disparities, given United States work in this area [32].

These actions would also probably help reduce ethnic inequalities in health, as (presumably) the type of mechanisms on the pathway from ethnicity to health are similar to those for the pathways from SEP to health (Figure 3). The important differences, though, are the role of racism and ethnicity and the mix of pathway mechanisms (eg, access to health services may be more relevant to ethnic inequalities in New Zealand [23,24]). Reducing discrimination could potentially assist in reducing smoking rates if the psychosocial stress associated with discrimination contributes to smoking given the New Zealand data on the adverse impacts of racism on health [33,34]. Also, more specific measures are required to continue to address past injustices (eg, through the Waitangi Tribunal). Fortunately, there is evidence that gaps between Māori and non-Māori are starting to decline for health, employment, educational and income achievement [4,35,36]. This may partly reflect specific policy initiatives and/or be an outcome of broad economic and social trends.

The need to reduce health inequalities attributable to smoking is recognised in the Ministry of Health's five-year plan for tobacco control which has specific targets for such inequalities [37]. Some of the specific interventions that could be considered are detailed below and some of these are already included in the Ministry of Health's plan:

### Enhanced tobacco regulation

There have been arguments in the New Zealand context for having a Tobacco Authority type agency [38] with a public health mandate to control the marketing of tobacco. This approach has also been proposed by others internationally [39,40]. Such an agency could allow the nicotine market to be realigned to strongly favour (in terms of price and availability) pharmaceutical-grade nicotine, over smoked forms of tobacco. Such a market could also favour reduced-harmed tobacco products such as nasal or oral snuff, though the idea of health sector endorsement of such a market is controversial in New Zealand. Nevertheless, a switch to snuff could plausibly facilitate reductions in overall harm to the health of users [41,42] and facilitate quitting [43,44] and therefore health inequalities attributable to tobacco use. Any shift to smokeless forms of nicotine or tobacco would also be likely to reduce the health inequalities associated with different levels of exposure to second-hand smoke. A key aspect for maximising the impact for reducing inequalities would be the extent to which the price differential could be managed given the suggestive evidence that low-income and Māori populations are more price sensitive (see the discussion around taxation below). This shift to alternate forms of nicotine or tobacco could also be accelerated by increasingly tight restrictions and raising the price of smoked tobacco.

In the long-term however, if a large proportion of low-income New Zealanders remained dependent on pharmaceutical nicotine or snuff – then this could still represent a drain on their financial resources (if the price was not kept relatively low). Such issues could be further explored by modelling work and studies on the price elasticities and acceptability of these alternative nicotine products to low-income New Zealanders.

Finally, enhancing tobacco regulation will inevitably be a political decision. Debate and discussion is therefore essential not only among the tobacco control community, but also politicians and the public at large. It is imperative that tobacco control advocates reinforce at all times that ridding New Zealand of tobacco smoking will benefit all sectors of society, *and *reduce inequalities – a win-win situation.

### Substantially enhanced comprehensive tobacco control policy

New Zealand could more intensively pursue all the key components of a comprehensive tobacco control programme (eg, tax policy, smokefree environments, and smoking cessation support – as detailed below). All these could be funded by increasing the relatively low level expenditure on tobacco control, currently less than 3% of tobacco tax revenue [45]. Added to these interventions could be litigation against the tobacco industry by government and a more strongly industry-focused approach to tobacco control [46].

### Tobacco taxation policy

There is some international evidence that tobacco taxes are relatively more effective in reducing tobacco consumption among low-income or poorly educated populations [47-49]. There are also some New Zealand data to support this differential benefit for low-income groups and Māori [50,51]. Other New Zealand modelling work [52] provides some justification for tobacco taxation, as it indicates that the harm from smoking for low-income New Zealanders greatly exceeds the likely harm from financial hardship that is associated with the tax. Despite this, there is concern regarding the potential for increased economic hardship (with subsequent impacts on health) among low-income groups from increased tobacco taxation in the future. If tobacco taxes were to be increased, it would be necessary and ethical [53] for a greater proportion of the tax revenue to be used for smoking cessation support, especially for Māori, Pacific and low-income New Zealanders. Indeed, this country previously introduced a programme of providing heavily subsidised nicotine replacement therapy in the year of the last tax increase (ie, 2001).

### Smokefree environments

In late 2004 a new smokefree law came into effect in New Zealand and the evidence to date is that it is working well [54-56]. This law covers all indoor workplaces and hospitality settings which suggests that it should reduce exposure to second-hand smoke among low-income workers and patrons of venues such as bars, clubs, and casinos. In addition, recent government-funded mass media campaigns may be contributing to increasing smokefree homes among low-income families (though post-campaign follow-up data have not yet been published). There is some indirect evidence for benefit from such campaigns internationally on smokefree homes [57].

The prevalence of smoking in cars amongst people from more deprived areas in New Zealand is significantly higher than less deprived areas [58]. While some campaigns have incorporated the hazard from smoking in cars in New Zealand, these have been of low intensity. Nevertheless, laws have now been passed in other jurisdictions (eg, Arkansas, Louisiana and Puerto Rico) and this approach could be considered in New Zealand.

### Mass media campaigns (smoking cessation)

There is evidence that mass media campaigns (both generic and those designed by Māori) are effective in stimulating calls to the national Quitline from Māori and other low-income New Zealanders who are the priority audiences for this service [59,60]. Evaluation work has also shown that culturally appropriate mass media campaigns are regarded as acceptable to a Māori audience [61,62]. A recently launched media campaign with a Pacific peoples focus [63] has also successfully stimulated increased call rates to the Quitline [Personal communication, Helen Glasgow, Director Quit Group, 28 March 2006].

### Smoking cessation services

The national free-phone Quitline service has been successful in reaching a Māori audience [60,64]. The popular uptake of heavily subsidised smoking cessation services provided via the Quitline [65] also suggests that it is reaching low-income New Zealanders. However, the idea of building long-term relationships with smokers and recruiting them as volunteers to promote smoking cessation services [66] has yet to be tried in this country.

Culturally appropriate smoking cessation services such as the Aukati Kai Paipa services for Māori women have been evaluated and found to be acceptable and effective [67]. New Zealand has also had some success with running quit and win contests [68] which may differentially appeal to low-income smokers (although this aspect has not been studied). A randomised controlled trial of bupropion for Māori smokers has reported successful smoking cessation outcomes in this population [69].

Other countries are also trying to reduce health inequalities associated with tobacco. A review of 16 studies that aimed to reduce smoking in low-income groups found that half of these had demonstrated effectiveness [70]. Out of another nine studies that were not actually targeted at low-income groups, in five of these the intervention was at least as effective in low as in high-income groups. Nevertheless, in four of the studies, including one New Zealand study [71], the intervention was less effective for those in low-income groups. In particular, there is evidence from the United Kingdom that smoking cessation services are reaching disadvantaged communities [72], that the services to such communities are qualitatively better [73], and that these services are reducing inequalities in smoking prevalence rates [74].

## Conclusion

There is extensive evidence that demonstrates that smoking prevalence is higher amongst those with low socioeconomic position (SEP), and Māori and Pacific peoples (compared to those of "New Zealand European" ethnicity) in the New Zealand setting. There are also many studies that indicate that the health burden attributable to tobacco is higher amongst these populations and that the associated relative health inequalities appear to be increasing. The estimated contributions of smoking to inequalities in mortality by SEP and ethnicity stand out relative to the many other drivers of health inequalities (ie, at 21% for SEP in men, and 8% for ethnicity in women). This should make the tobacco contribution worthy of the attention of policymakers, especially given the evidence for the effectiveness and cost-effectiveness of tobacco control interventions. Another reason for attention to this role for tobacco is the likelihood that, under business as usual, tobacco will probably grow in importance as a contributor (in relative terms) to health inequalities. Besides the ethical arguments for reducing inequalities to achieve justice, there are additional arguments in New Zealand for such actions. These include obligations under the Treaty of Waitangi for the government and the health sector and the need to address past harms associated with colonisation.

There is some evidence from New Zealand and elsewhere for health sector actions that reduce smoking by low-income populations and indigenous peoples. These include tobacco taxation, thematically appropriate mass media campaigns, and appropriate smoking cessation support services. There is however, major scope for improvements in tobacco regulation and better resourcing of a more intensive and comprehensive tobacco control programme in this country. In particular, there is potential for a tighter regulatory framework that could rapidly shift the nicotine market towards pharmaceutical-grade nicotine (or smokeless products such as snuff) and away from smoked tobacco.

**Disclaimer: **The views of the authors do not necessarily reflect the views of their employing organisations – including the New Zealand Ministry of Health.

## Competing interests

The author(s) declare that they have no competing interests.

## Authors' contributions

This article was conceived by TB and MT. The first draft was written by NW and TB. All authors contributed to subsequent drafts and approved the final manuscript.
