# Correction: what potential has tobacco control for reducing health inequalities? The New Zealand situation

**DOI:** 10.1186/1475-9276-5-16

**Published:** 2006-12-18

**Authors:** Nick Wilson, Tony Blakely, Martin Tobias

**Affiliations:** 1 Department of Public Health, Wellington School of Medicine & Health Sciences, University of Otago, PO Box 7343, Wellington South, New Zealand; 2 Ministry of Health, PO Box 5013, Wellington, New Zealand

## Abstract

This is a correction article.

## Text

In our article [1] there was an error in the calculation of population attributable risk percents (PAR%) for 1996–99 shown in Table 1 (bottom row). The corrected Table is in this correction article (see Table 1). This correction has also required revising Figure 2 (see Figure 1 in this correction article). In the process of making these corrections, we have extended our presentation of the contribution of smoking to mortality gaps by ethnicity and education to include a more explicit acknowledgement of the choice of counterfactual assumption. Figure 1 now shows the estimated 'never smoker rate' plus the 'smoking attributable rate' for each ethnic and educational group. Note that the 'smoking attributable rate' as a percentage of the total rate is equivalent to the relevant PAR% shown in Table 1. 'A' and 'B' signify two alternative counterfactual scenarios that can be used to estimate the contribution of smoking to ethnic or socioeconomic gaps in mortality. Scenario A for ethnic gaps is whereby the non-Māori non-Pacific (nMnP) population adopt the smoking rates of Māori, calculated using direct standardisation as given elsewhere [ref 27 of the original paper]. Scenario A for education gaps is whereby each educational group is given an 'average' smoking rate, calculated using Poisson regression as given elsewhere [ref 75 of the original paper]. Scenario B is more extreme (and arguably somewhat unrealistic) whereby we assume there had never been smoking in New Zealand, with the area labelled 'B' in Figure 1 being that for Scenario B over and above that for Scenario A. The contribution of smoking to gaps under Scenario B is calculated using standard population attributable rate methods, that is the difference in "attributable smoking rates" between Māori and nMnP or between nil and post-school qualifications. Thus estimating the contribution of smoking to mortality gaps depends on how extreme the counterfactual assumptions are [2]. Halving total population smoking rates, and making smoking rates for all ethnic and socioeconomic groups the same, might (allowing for time lags) close mortality gaps by an amount mid-way between Scenarios A and B shown in Figure 1.

**Table 1:** (Corrected): The estimated percentage decrease (population attributable risk percent (PAR%)) in 45–74 year old mortality rates during 1996–99 had all current and ex-smokers actually been never smokers

	**Men 1996–99**				**Women 1996–99**			
	
***Within educational group †***	**PAR% in total population**	**PAR% within educational group**			**PAR% in total population**	**PAR% within educational group**		
				
		**Nil**	**School**	**Post-school**		**Nil**	**School**	**Post-school**
(ii) All current and ex-smokers become never smokers in each educational group (ie, historically smokefree).	26%	29%	26%	23%	25%	27%	24%	23%
	
***Within ethnic group ‡***	**PAR% in total population**	**PAR% within ethnic group**			**PAR% in total population**	**PAR% within ethnic group**		
				
		**Māori**	**nMnP**			**Māori**	**nMnP**	

(ii) All current and ex-smokers become never smokers in each ethnic group (ie, historically smokefree).	25%	17%	28%		24%	25%	25%	

nMnP – non-Māori non-Pacific (ie, mainly "New Zealand European" ethnicity). See the footnotes to Figure 1 in the original article for ethnicity definitions.

† Source: Table 4 of reference 75 in the original article.

‡ Source: PAR% calculated from data in reference 27 in the original article.

NB: The educational PAR% estimates are calculated using Poisson rate ratios adjusted for age and ethnicity, whereas the ethnic PAR% estimates are based on age-standardised mortality rate

**Figure 1:**
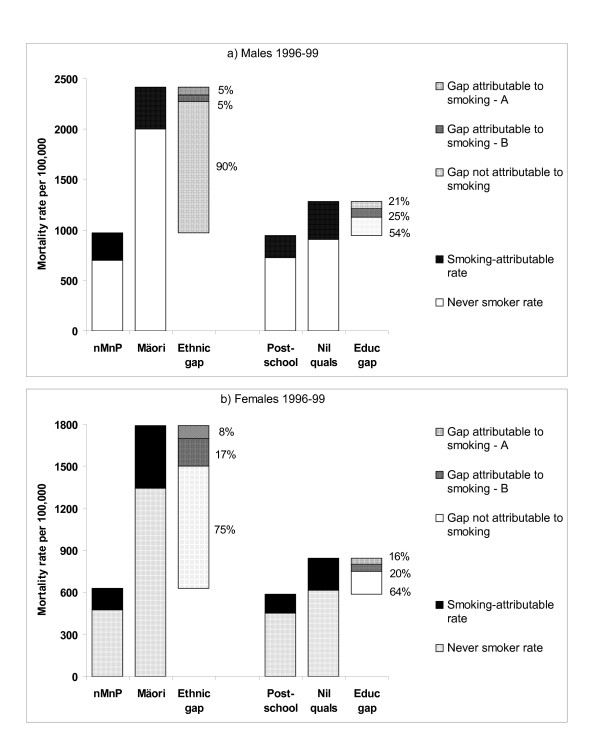
**(Corrected version of Figure 2): The contribution of active tobacco smoking to 45–74 year old age-standardised mortality rates, and gaps in mortality rates, in 1996–99, by ethnicity and education (with the latter as a marker for SEP)**. nMnP – non-Māori non-Pacific (ie, mainly "New Zealand European" ethnicity). The percentage labels give the percentage contribution of smoking to gaps for Scenario A and the added contribution of Scenario B (see text in this correction article for more details).
